# Hsp70 proteins in mitosis and disease

**DOI:** 10.18632/oncotarget.5965

**Published:** 2015-10-04

**Authors:** Laura O'Regan, Josephina Sampson, Andrew M. Fry

**Affiliations:** Department of Molecular and Cell Biology, University of Leicester, Leicester, UK

**Keywords:** mitosis, Hsp70, Hsp72, Nek6, K-fibre

Heat shock proteins (HSPs) are ATP-dependent molecular chaperones which aid folding of nascent polypeptides, maintain proteins in unstable conformations and prevent protein denaturation. These functions are essential in many biological contexts, including assembly and disassembly of macromolecular complexes, trafficking of proteins and regulation of enzyme activity [1]. Mitotic cell division is particularly complex involving rapid changes in cytoskeletal and organelle architecture. One would therefore expect it to be highly dependent on HSPs; however, much remains to be learnt about the roles of HSPs in mitosis.

In a recent study, we discovered that Hsp72, an inducible cytoplasmic isoform of the Hsp70 family, is essential to build a mitotic spindle capable of efficient chromosome congression and segregation [2]. Firstly, Hsp72 contributes to generation of stable kinetochore (K)-fibres. K-fibres are bundles of microtubules that connect the spindle poles with the kinetochores and are essential for chromosome movement. Upon depletion of Hsp72 or addition of an Hsp70 inhibitor, cells exhibited reduced K-fibres. This was coincident with misaligned chromosomes, metaphase delay and a strongly active spindle assembly checkpoint. The loss of K-fibres was not caused by reduction in microtubule nucleation, but rather failure to recruit the K-fibre-stabilising proteins, ch-TOG and TACC3. Hsp72 localises to spindle poles and spindle fibres in a similar manner to ch-TOG and TACC3. Furthermore, when TACC3 was immunoprecipitated from cells treated with the Hsp70 inhibitor, association with its partner, ch-TOG, was reduced. Together, this suggests that Hsp72 stabilises K-fibres by facilitating assembly of ch-TOG and TACC3 into a complex that can then serve to bundle K-fibre microtubules [3].

Secondly, abrogation of Hsp72 function led to reduced interpolar distances, reduced astral microtubules and misoriented spindles. Astral microtubules attach the spindle to the cell cortex to maintain its shape and position. These data indicate that Hsp72 has additional functions in astral microtubule organization or cortical attachment. This is likely to be independent of the ch-TOG-TACC3 complex as this is not thought to be required for astral microtubule function. Importantly, whilst Hsp72 depletion and chemical inhibition of Hsp70 give similar phenotypes, there are some clear differences. This can be explained by that fact that chemical inhibition blocks the activity of all Hsp70 isoforms and it is possible that other Hsp70 isoforms have roles in mitosis. On the other hand, chemical inhibition does not remove the protein and so the different phenotypes may result from Hsp72 having mitotic functions as a scaffold that are independent of its catalytic activity.

Hsp72 is phosphorylated in mitosis by Nek6, a protein kinase that is also required for robust spindle assembly [4, 5]. Nek6 phosphorylates Hsp72 on T66, a residue that sits within the nucleotide-binding domain just upstream of the catalytic lysine (K71). Mislocalization of a T66A mutant and failure of Hsp72 to associate with the spindle in the absence of Nek6 indicate that phosphorylation mediates localisation of Hsp72 to the spindle. Furthermore, expression of the T66A mutant resulted in reduced K-fibres, whilst the T66E mutant could rescue the K-fibre defects and loss of ch-TOG/TACC3 recruitment to K-fibres seen upon either Hsp72 or Nek6 depletion. However, understanding the mechanism through which phosphorylation regulates Hsp72 will require molecular insights on Hsp72 interactions with its mitotic partners.

Interestingly, a phosphospecific antibody revealed that Hsp72 phosphorylated on T66 was not only localised to the spindle apparatus, but also enriched on spindle poles and kinetochores. This begs the question of whether phosphorylated Hsp72 has additional, perhaps distinct, substrates at the kinetochore. The absence of total Hsp72 may lead to loss of function of these proteins as well, exacerbating the K-fibre assembly and chromosome congression defects. Similarly, problems in cortical attachment of microtubules could contribute to loss of astral microtubules and spindle orientation defects. It will thus be important to ascertain whether (phosphorylated) Hsp72 may stabilize the plus ends of microtubules that contact kinetochores and the cell cortex.

Besides their homeostatic function, HSPs have an important role in protecting cells from the proteotoxic stress that can arise in different pathological states [6]. These include protein-folding disorders, autoimmune diseases and cancer. In cancer, the induced expression of Hsp70 due to proteotoxic stress promotes cell survival and tumor progression. As cancer cells undergo frequent mitotic division, Hsp70 inhibitors could have therapeutic value in targeting the proliferating tumour tissue. Whilst it has been frustratingly difficult to develop potent and selective inhibitors of Hsp70 itself [7], drugging upstream regulators, such as Nek6, offers an alternative approach. In contrast to cancer, Hsp70 proteins are beneficial to the patient in slowing the onset of neurodegenerative disorders, such as Alzheimer's, Huntington's or Parkinson's disease. Here, they promote removal of misfolded proteins through autophagy or proteasomal degradation. However, as neuronal cells are post-mitotic and do not undergo division, anti-cancer therapies specifically targeting the mitotic form of Hsp70 would have the advantage of not precipitating the onset or progression of neurodegenerative diseases.
